# Case report: MRI of the brain in metronidazole toxicity

**DOI:** 10.4103/0971-3026.69355

**Published:** 2010-08

**Authors:** Vishal Kalia, Vibhuti, Kavita Saggar

**Affiliations:** Department of Radiodiagnosis, Dayanand Medical College & Hospital, Ludhiana – 141 001, India

**Keywords:** Dentate nuclei, diffusion restriction, metronidazole toxicity, MRI

## Abstract

Metronidazole is a commonly used antimicrobial drug. When used excessively, it can cause encephalopathy. We report the MRI findings in one such case. A 43-year-old male patient was treated with metronidazole for 2 months, for an amebic liver abscess and presented with neurological signs and symptoms. MRI of the brain showed findings consistent with metronidazole toxicity.

## Introduction

Metronidazole is a commonly used antibiotic for various anerobic and protozoal infections and is also used in Crohn disease. Inappropriate usage in excessive doses can give rise to neurological problems such as ataxia, seizures, peripheral neuropathies, cerebellar signs and symptoms, and encephalopathy. The neurological features usually become apparent when the drug is used in a dose exceeding 2 g/day for prolonged periods.[1]

## Case Report

A 43-year-old male presented with slurring of speech, generalized weakness, vertigo, and ataxia for 4 days. He had no history of headache, loss of consciousness, or seizures. His past history revealed that he had been admitted to a hospital 2 months earlier with abdominal complaints and had been diagnosed as a case of amebic liver abscess. At that time, USG-guided aspiration of the abscess had been done and serology for *Entamoeba histolytica* had been positive. He had been on metronidazole 400 mg orally thrice a day since then.

**Figure 1:**
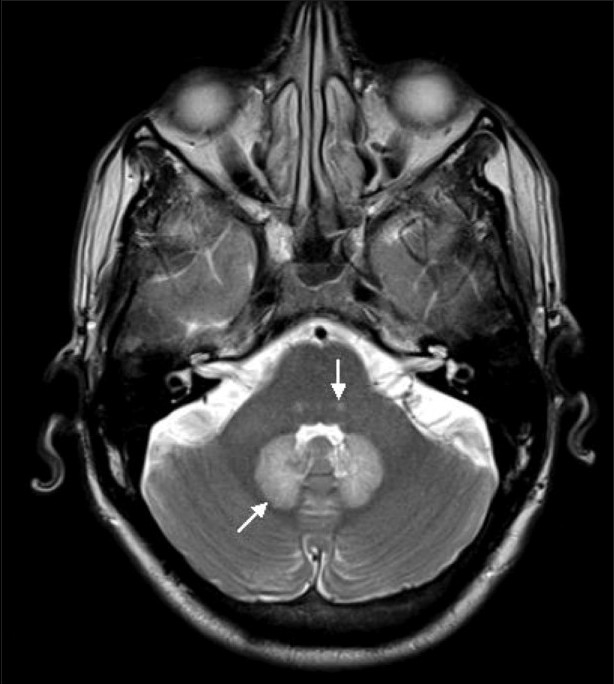
Axial T2W MRI of the brain shows symmetric areas of hyperintensity involving the dentate nuclei (arrow) and dorsal pons (arrowhead)

**Figure 2:**
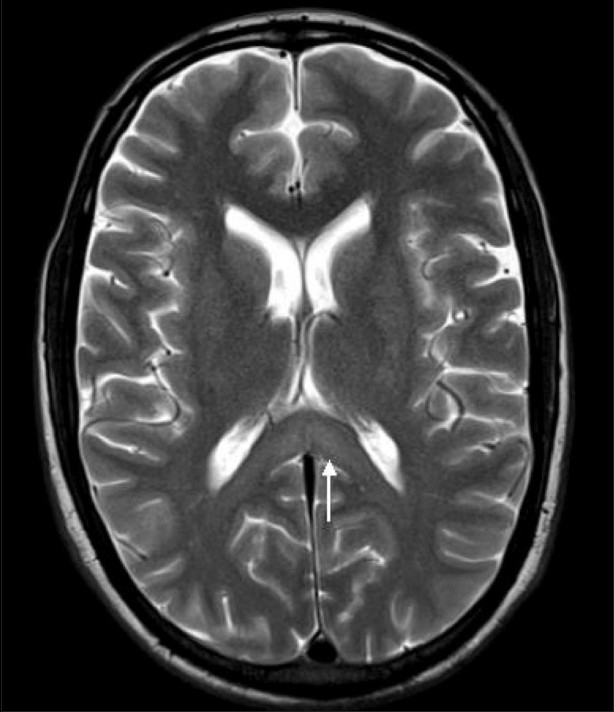
Axial T2W MRI of the brain shows symmetric areas of hyperintensity involving the splenium of the corpus callosum (arrow)

**Figure 3:**
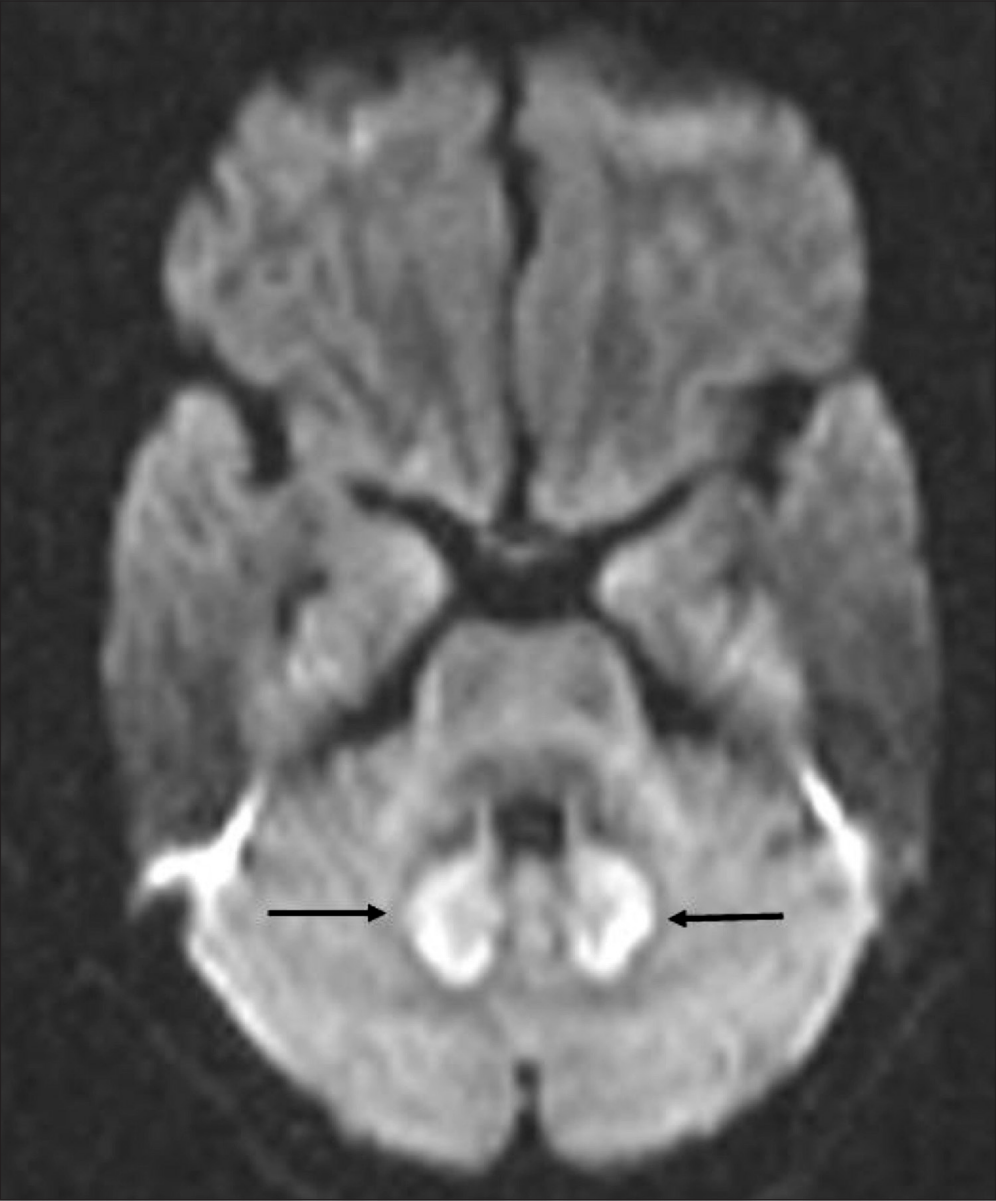
Axial diffusion weighted MRI image shows diffusion restriction in the dentate nuclei (arrow) and dorsal pons (arrowhead)

**Figure 4:**
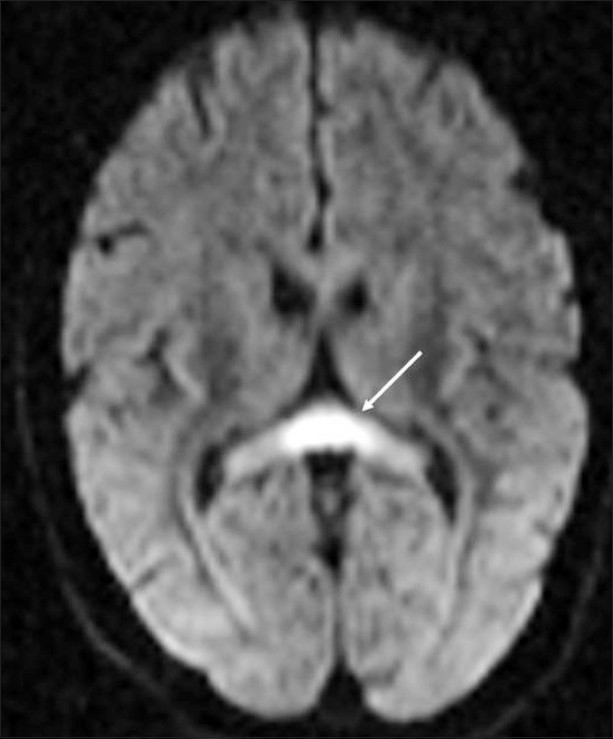
Axial diffusion weighted MRI image shows diffusion restriction in the splenium of the corpus callosum (arrow)

His neurological examination revealed horizontal nystagmus to the left and positive cerebellar signs. Liver function tests were mildly deranged (SGOT 73 IU/L, SGPT 72 IU/L, alkaline phosphatase 111 IU/L). Cerebrospinal fluid (CSF) examination showed 2 cell/mm^3^ with glucose of 37 mg/dL, proteins 33 mg/dL, chloride 121 mmol/L and was negative for cryptococcal antigen. MRI of the brain showed symmetrical areas of altered signal intensity, appearing hyperintense on T2W and fluid-attenuated inversion-recovery images and involving the dentate nuclei, dorsal pons, and splenium of the corpus callosum [Figures 1 and 2]. There was evidence of diffusion restriction on diffusion-weighted/apparent diffusion coefficient mapping [Figures 3 and 4]. No evidence of hemorrhage was seen on gradient recovery echo images. Based on the prolonged history of metronidazole intake, we considered the possibility of metronidazole toxicity. The patient was advised discontinuation of metronidazole and on follow-up he showed resolution of symptoms. Unfortunately, follow-up imaging is not available.

## Discussion

The exact incidence of metronidazole-induced encephalopathy is unknown and the underlying mechanism for brain injury has not been completely understood. It was Ahmed *et al*. who first described the imaging findings of metronidazole toxicity. They reported a 45-year-old female whose MRI brain showed symmetrical, bilateral, abnormal hyperintense signal in the supratentorial white matter, corpus callosum, and within the cerebellum and deep cerebellar nuclei on T2W images.[2] They suggested “axonal swelling with increased water content” due to toxic injury as the possible mechanism or, alternatively, localized reversible ischemia due to vascular spasm. Other theories that have been put forward include: (a) interstitial edema and ischemia manifesting as increased signal intensity on diffusion-weighted and apparent diffusion coefficient mapping or (b) Purkinje cell damage after high dose of metronidazole due to binding of the drug to neuronal RNA, causing inhibition of protein synthesis and resulting in axonal degeneration.[2]

MRI plays an important role not only in the diagnosis of this entity but also in the follow-up of these cases, where it may be of use in predicting the outcome after drug discontinuation. MRI may reveal increased signal intensity on T2W/fluid-attenuated inversion-recovery images in the cerebellar dentate nuclei, midbrain, dorsal pons (the vestibular nucleus, abducens nucleus, and superior olivary nucleus), splenium of the corpus callosum, and the dorsal medulla. Unusual sites are the inferior olivary nucleus and cerebellar white matter. According to Seok *et al*.,[3] in addition to the above mentioned abnormalities, increased signal intensity may also be seen in the anterior commissure and bilateral inferior olivary nuclei with hypertrophic change. These changes were not seen in our patient.

In their patient, after discontinuation of therapy, all the lesions improved, except for the hypertrophic change in the inferior olivary nuclei; this change may have been be due to interruption in the circuit of the Guillain-Mollaret triangle rather than metronidazole toxicity.[3] High signal intensity on diffusion-weighted and apparent diffusion coefficient map values may indicate that cytotoxic edema is the underlying cause and, depending on the apparent diffusion coefficient map values, we can predict the tissue viability and hence the severity and reversibility of metronidazole-induced encephalopathy.[3,4]

In our case, the diagnosis of metronidazole toxicity was made clinically and was supported by the MRI findings.

The differential diagnosis includes demyelinating diseases, and metabolic, infectious, and inflammatory processes. Multiple sclerosis and acute disseminated encephalomyelitis may present with similar MRI findings, but involvement of the gray matter, a normal CSF, and the temporal profile make these possibilities unlikely. Wernicke encephalopathy is another differential diagnosis, but the involvement is predominantly of the diencephalon and midbrain.[2] Atypical non-alcoholic Wernicke’s encephalopathy can sometimes present with MRI findings of T2 hyperintensity involving the dorsal medulla and cerebellum.[1] Heat stroke can also rarely involve the cerebellum but the predominant involvement is of the thalami and external capsules.[5] The differential diagnosis of T2 hyperintense, bilaterally symmetrical dentate nuclei includes methyl bromide intoxication, maple syrup urine disease and enteroviral encephalomyelitis.[6] The differential diagnosis of T2 hyperintense lesions in the splenium of the corpus callosum includes Marchiafava-Bignami disease, encephalitis (demyelinating, influenza, *Escherichia coli*, mumps, adenovirus, Epstein-Barr virus and Rota virus), osmotic myelinolysis, acute toxic encephalopathy and anti-epileptic drugs.[6–8]

However, despite the long list of possible differential diagnoses, toxicity in cases can be suspected in the presence of the characteristic MRI distribution of lesions, i.e., bilaterally symmetric, with involvement of the cerebellar dentate nuclei in most cases, and confirmed when the characteristic history of metronidazole intake is available and there is reversal of symptoms and MRI findings after cessation of drug intake. Drug toxicity should be included in the differential diagnosis when the MRI pattern reveals multifocal disease, with little or no mass effect.[2]
